# Human gait-labeling uncertainty and a hybrid model for gait segmentation

**DOI:** 10.3389/fnins.2022.976594

**Published:** 2022-12-08

**Authors:** Jiaen Wu, Henrik Maurenbrecher, Alessandro Schaer, Barna Becsek, Chris Awai Easthope, George Chatzipirpiridis, Olgac Ergeneman, Salvador Pané, Bradley J. Nelson

**Affiliations:** ^1^Multi-Scale Robotics Lab, ETH Zurich, Zurich, Switzerland; ^2^Magnes AG, Zurich, Switzerland; ^3^Cereneo Foundation, Center for Interdisciplinary Research (CEFIR), Vitznau, Switzerland

**Keywords:** gait labeling uncertainty, limit of agreement, convolutional neural network, dynamic time warping, automatic gait segmentation, wearable inertial sensors, gait event detection, human activity recognition (HAR)

## Abstract

Motion capture systems are widely accepted as ground-truth for gait analysis and are used for the validation of other gait analysis systems. To date, their reliability and limitations in manual labeling of gait events have not been studied.

**Objectives:**

Evaluate manual labeling uncertainty and introduce a hybrid stride detection and gait-event estimation model for autonomous, long-term, and remote monitoring.

**Methods:**

Estimate inter-labeler inconsistencies by computing the limits-of-agreement. Develop a hybrid model based on dynamic time warping and convolutional neural network to identify valid strides and eliminate non-stride data in inertial (walking) data collected by a wearable device. Finally, detect gait events within a valid stride region.

**Results:**

The limits of inter-labeler agreement for key gait events heel off, toe off, heel strike, and flat foot are 72, 16, 24, and 80 ms, respectively; The hybrid model's classification accuracy for stride and non-stride are 95.16 and 84.48%, respectively; The mean absolute error for detected heel off, toe off, heel strike, and flat foot are 24, 5, 9, and 13 ms, respectively, when compared to the average human labels.

**Conclusions:**

The results show the inherent labeling uncertainty and the limits of human gait labeling of motion capture data; The proposed hybrid-model's performance is comparable to that of human labelers, and it is a valid model to reliably detect strides and estimate the gait events in human gait data.

**Significance:**

This work establishes the foundation for fully automated human gait analysis systems with performances comparable to human-labelers.

## 1. Introduction

Human walking is a complex dynamic process. It integrates multiple interdependent sensory inputs and requires coordination of the motor outputs to achieve efficient, stable, and adaptive locomotion. The deterioration of human gait can severely affect people's mobility, reduce a person's level of activity, and affect their quality of life. In general, gait can be described by its temporal and spatial characteristics such as stride time, stride length, stride velocity, swing time, stance time, and their corresponding variability as well as left and right symmetry metrics (Del Din et al., 2016). Probing and evaluating these gait characteristics is important not only in the field of rehabilitation (Richards et al., 1999; Eng and Tang, 2007; Selves et al., 2020), but also in the early detection of neurological diseases, such as Parkinson's disease (Lees, 1992; Brognara et al., 2019), Alzheimer's disease (Gras et al., 2015; Mc Ardle et al., 2019), and tremor syndromes (Stolze et al., 2001; Hoskovcová et al., 2013). Some specific gait patterns even appear in the earliest stage of neurological diseases before other signs and symptoms are evident, providing significant clues for early diagnosis and treatment (Jacobi et al., 2012; Nürnberger et al., 2015; Pistacchi et al., 2017). Therefore, quantitative gait characteristics can be used as a digital biomarker for monitoring and assessing people's health status.

To assess and evaluate an individual's gait by temporal and spatial characteristics quantitatively, gait is segmented into repeating cycles. Each cycle starts when the foot leaves the ground commencing the swing phase and is followed by the stance phase, where the foot is on the ground again, which lasts until the next gait cycle starts (DeLisa, 1998). These two main phases can be further divided into eight sub-phases by gait events within one gait cycle: initial contact, loading response, mid-stance, terminal stance, pre-swing, initial swing, mid-swing, and terminal swing. Key gait events segmenting these phases are heel strike (HS) and toe off (TO). Additional events of interest include heel off (HO) and flat foot (FF), which give insights into the transition periods between the two main gait phases, swing and stance. For pathological gait, the preceding sequence and the time allocation of gait phases can vary compared to healthy people, and some gait events or phases might even be missing completely (Senanayake and Senanayake, 2010; Meng et al., 2013). Observing these variations in gait events long-term, during daily activities provides a powerful tool for clinical gait assessment, evaluation, and diagnosis (Atrsaei et al., 2021), such as identifying early Parkinson's disease (PD) (Rehman et al., 2019), capturing freezing of gait (Palmerini et al., 2017; Mancini et al., 2019), monitoring PD symptoms (Heijmans et al., 2019), and the Levodopa response (Pulliam et al., 2017). In light of the ongoing shift of rehabilitation and early diagnosis from physical visits of care facilities toward remote monitoring systems, the understanding of real-world gait data gains significant importance (Warmerdam et al., 2020). Recent studies have investigated the relationships between clinical gait assessments and home-based monitoring systems, and have described an additive component for real-world data (Shah et al., 2020; Atrsaei et al., 2021). A reliable method for continuous remote monitoring of gait quantity and quality affords new perspectives of assessing the real-world impact of interventions and of investigating the transfer of rehabilitation progress to out-patient behavior.

To capture the quality of gait, a significant research effort has been made toward achieving a comprehensive understanding of spatio-temporal gait characteristics, with the aid of various systems such as motion capture systems (Mihradi et al., 2011; Carse et al., 2013), pressure mats (Yu et al., 2010; Papavasileiou et al., 2017), and wearable sensors (Tunca et al., 2017; Zhang et al., 2019; Wu et al., 2021; Celik et al., 2022). Compared to traditional subjective visual observations by therapists, instrumented assessments allow for an objective assessment of gait characteristics (Laughman et al., 1984). Motion capture systems give a detailed evaluation of motion but require a complex stationary set-up involving cameras and the collected data needs to be post-processed (manually) to identify relevant gait characteristics. This makes those gait systems very expensive to operate. Moreover, motion capture systems cannot track people outside the laboratory and therefore the time and place of the data collection are severely constrained as the patient must physically visit a gait laboratory. An alternative is pressure mats, which measure the interface pressure when people are treading on them. While the setup has reduced complexity, gait measurement is still confined to a gait laboratory and the available range of gait parameters and information from pressure mats is limited. Both motion capture systems and pressure mats offer a constrained walking environment, allowing individuals to focus their attention solely on ambulation. This may lead to biased results, as the recorded gait is not representative of the patient's typical gait. Additionally, the measurements often take place under the supervision of a health professional, which may further skew the collected data (Kaye et al., 2012; Robles-Garćıa et al., 2015). These costly and laboratory-limited data acquisition systems have led to a growing urge for cheaper and mobile systems, i.e., wearable gait analysis system (WGAS) (Tunca et al., 2017; Zhang et al., 2019; Wu et al., 2021). WGAS are not only cost-effective and portable, but can also be used for a longer period of time of continuous monitoring, allowing for unbiased measurements during daily routines.

For validation of WGAS's algorithms, optical motion capture systems are the most commonly used reference systems and are considered as the gold standard (Caldas et al., 2017; Kobsar et al., 2020). They employ either retro-reflective (passive) or infrared emitting (active) markers attached to different locations on the lower limbs. These markers are tracked through multiple cameras to record their spatial trajectories. The key gait events are then identified by technicians who visually examine the markers' spatial trajectories or by automated event detection algorithms based on markers' position and velocity (Kidziński et al., 2019; Lempereur et al., 2020) for further gait parameter estimation. Although the aforementioned automated detection algorithms are time efficient, they are not suited for the patient population due to the high variability that is intrinsic to pathological gait. Thus, the manual labeling of motion capture system data is still the most widely accepted benchmark for validation of WGAS algorithms for gait analysis (Caldas et al., 2017; Tunca et al., 2017; Hsu et al., 2018). Nonetheless, the uncertainty of manual labels of motion capture system data and the limit of detection have never been considered and evaluated. The uncertainty in manual labels mainly comes from two sources: 1) the lack of consistency within a labeler i.e., intra-labeler inconsistency; and 2) the lack of consistency among different labelers i.e., inter-labeler inconsistency. The label quality of gait events and acquisition results are highly dependent on the labeler's personal perspective and experience level. The uncertainty of labels in gait data resulting from intra-labeler and inter-labeler inconsistency can have a direct impact on the reliability of gait event labels, and further affect the accuracy of gait parameter estimation and the decision by doctors. Therefore, one objective of this study is to investigate the uncertainty in manual labeling of gait events in the well-accepted benchmark, i.e., optical motion capture system data.

To estimate spatial and temporal gait parameters from WGAS, different algorithms have been developed. The first group of gait analysis methods relies on straight gait event detection in long-term sensor signals (Agostini et al., 2013). Gait events can be identified by different techniques, including rule-based (Zhu et al., 2012; Zhao et al., 2017) and machine learning methods (Zhen et al., 2019; Liu et al., 2021). The second group of methods splits gait analysis into two stages: first segmenting the sensor data into strides, and then searching for gait events within each found stride. The advantages of identifying single strides first are that it is more robust for pathological gait where some gait events may be missing (Agostini et al., 2013), and more efficient for long-term gait recording processing since it rejects non-stride data in the first stage, reducing the computational cost (Ullrich et al., 2020). This two-stage gait analysis has been investigated in literature by many techniques, such as threshold/peak detection methods (Hickey et al., 2016; Ullrich et al., 2020), template matching methods (Barth et al., 2015; Oudre et al., 2018) and machine learning-based methods (Martindale et al., 2021). However, robust algorithms for automatic detection of stride and rejection of non-stride data from long-term free-living walking data are still needed for effective gait analysis. Therefore, in this paper, a hybrid approach based on dynamic time warping (DTW) and convolutional neural network (CNN) is proposed to automatically identify stride signals and eliminate non-stride signals from long-term inertial walking data.

The contribution of this study is two-fold: 1) The uncertainty in terms of the limit of agreement (LOA) in manual labeling of human walking data from an optical motion capture system is assessed and analyzed. Those manual labels are further used as benchmarks for the validation of the proposed gait analysis algorithm. Their inherent variances serve as a baseline for the validation study. 2) A hybrid approach based on DTW and CNN named *stepperNet* is developed for gait cycle identification in long-term walking. A gait event detection algorithm is later employed to segment each stride data-slice into sub-phases. The results of gait event detection are compared to the average manual labels obtained from the work done in contribution 1).

## 2. Materials and methods

### 2.1. Data acquisition

#### 2.1.1. Participants of the study

Three groups $G_{i}$ with *i* ∈ {1,2,3} of healthy participants were recruited in this study. The first group $G_{1}$ consisted of four healthy participants (all males, with an average age of 39 ± 7.07 years), who were instructed to walk on a treadmill. They are labeled as participant *S*_1_ − *S*_4_. The dataset of this group was used for the assessment of manual labeling and validation of gait event detection algorithm. The second group $G_{2}$ consisted of (distinct) four healthy participants (all males, with an average age of 31.5 ± 2.06 years), who were instructed to walk for several minutes at various gait regimes. They are labeled as participant *S*_5_ − *S*_8_. The dataset of this group was used for *stepperNet* training and validation. The third group $G_{3}$ consisted of another five labelers (four males and one female, with an average age of 29.6 ± 1.67 years). Note that the $G_{3}$ is not a walking group but a group of labelers. To minimize the effect of different labeling experience of labelers and to keep them with the same level of labeling experience, the five labelers were well-trained for the same amount of time to manually label the gait events on the software of the motion capture system. They are labeled as participant *S*_9_ − *S*_13_.

All participants of $G_{1}$ and $G_{2}$ had no known injuries or abnormalities that affected their gait and were asked to walk as much as possible in the same way they walk in their daily lives. Written informed consent was provided by all participants and this study was conducted in accordance with Good Clinical Practice guidelines and the Declaration of Helsinki after receiving a declaration of clearance from the local ethics committee (BASEC Nr Req-2019-00715).

#### 2.1.2. Wearable gait analysis system

**Figure 1:**
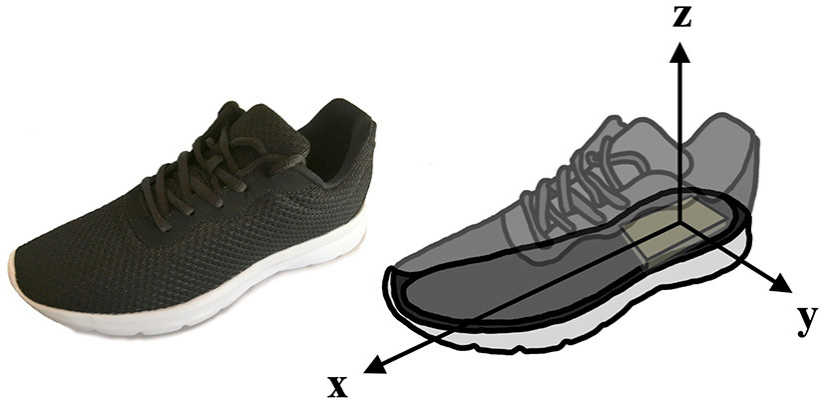
Schematic illustration of WGAS ecosystem embedding sensors in the outsole of the shoes.

Gait data were recorded with the WGAS as shown in Figure 1. It consists of a pair of shoes with custom-developed embedded electronics. The electronics include a wireless-enabled (BLE and Wi-Fi), a dual-core MCU (ESP32, Espressif Systems CO. Ltd., Shanghai, China) running a custom FreeRTOS-based^1^ C firmware for handling communication with a custom iOS application, and on-board sensors (LSM6DSM and LSM303AGR, STMicroelectronics, Geneva, Switzerland). The system allows logging sensor data to a local SD card. The WGAS also includes vibration-motors for haptic-feedback.

According to the needs of this research, the range of the accelerometer and gyroscope are set to ±8*g* and ±2, 000°/s, respectively. The sampling frequency of data acquisition is set to 100 Hz. The sensor's body frame, in which the acceleration, angular rate, and magnetic field are measured, can be seen in Figure 1. The *x*−axis is aligned with the shoe's major axis from heel to toe (pointing forwards), while the *z*−axis is normal to the shoe's sole and pointing upwards (toward the ankle and leg of the wearer). Lastly, the *y*−axis is set in accordance to *x* and *z*, such that the frame is right-handed.

#### 2.1.3. Data acquisition and notation

**Data acquisition for** $G_{1}$ The participants of the first group $G_{1}$ were instructed to wear the WGAS and walk with a self-selected gait on a treadmill. They were guided to complete three trials without assistance (e.g., handrail or body weight support) at *N*_*v*_ = 3 different walking speeds, specifically 0.53, 0.86, and 1.11*m*/*s*. The treadmill (Bertec) was equipped with two force plates beneath the treadmill belt measuring ground reaction force sampled at 600 Hz. Retroreflective markers (marker size 14 mm) were placed on participants' shoes at the toes, the first and fifth Metatarsophalangeal joints, and the calcaneal tuberosities, as well as on the medial and lateral malleolus of the ankle, and on their hands. The markers on the hands were used to identify the hand clapping movements performed by the participants, which were used to signal the start of steady-state walking in the experiment. Positional data of markers were collected by the motion capture system (Qualisys Opus 400 with 9 cameras) running at 100 Hz.

The data-acquisition protocol involved the following stages:

1. Data-logging was enabled for both systems, the WGAS and the motion-capture and force-plate system.
2. A simple calibration process was carried out before each walking trial began, consisting of turning-on and -off of the vibration on both shoes to give a signal for data synchronization. The vibration was registered by the WGAS as a binary signal (ON/OFF) and by the treadmill's force-plates. The registered vibration signals allowed for synchronization of the motion capture data and the WGAS data in post-processing.
3. The treadmill was subsequently ramped up to the respective speed and the participants were asked to acquaint themselves with the speed.
4. Once the treadmill reached the correct speed and the participants were comfortable walking at its speed, they performed a pronounced clapping motion with their hands to indicate the start of the steady-state motion capture measurement.
5. Participants then walked for approximately *N*_*s*_ = 50 strides for left and right foot, respectively. Stride count was monitored by a dedicated person.
6. After completing 50 strides on each side, the motion capture measurement was terminated, followed by a stop of the WGAS logging and a halting of the treadmill.

This protocol was repeated for each participant and speed.

Data obtained from the motion capture system contained the markers' positional data in three orthogonal axes, and the magnitude of the exerted force on the treadmill including its orientation vector in three axes. Data obtained from the WGAS consisted of acceleration, angular velocity and magnetic field vectors in three axes.

**Data acquisition for** $G_{2}$ Each individual of the second group of participants $G_{2}=\left\{S_{5},\ldots ,S_{8}\right\}$, was instructed to wear the WGAS and collect three distinct datasets. The first was a 2-min recording of their normal gait at their preferred walking speed. The second dataset consisted of the participant imitating various walking impairments, including spastic gait, steppage gait and waddling gait for another 2 min. Thirdly, they were asked to collect any range of non-stride motions, such as stomping, jumping, backwards and sideways walking, etc. The three data recordings were collected outdoors.

**Notation** Let $a_{x}\in {\mathbb{R}}^{m}$ denote a column vector consisting of the sensor's acceleration data in *x* axis, where ***a*****_*x*_**[*k*] denotes the *k*-th entry of ***a*****_*x*_** and is the acceleration data at time stamp *k*, *m* ∈ ℕ denotes the length of the acceleration data, i.e., the number of collected samples. Similarly, ***a*****_*y*_** and ***a*****_*z*_** are column vectors of the sensor's acceleration data in *y* and *z* axis, respectively. Analogously, ***ω*****_*x*_**, ***ω*****_*y*_**, and ***ω*****_*z*_** denote the data column vectors of angular velocity. Given any data vector, here we use the accelerometer signal ***a***_*x*_ as an example, $a_{x}\in {\mathbb{R}}^{m}$, $\bar{E}\left(a_{x}\right)=\sum_{k=1}^{m}\frac{a_{x}\left[k\right]}{m}$ denotes its sample mean. Time derivative signals of ***a***_*x*_ are denoted and computed as ${\dot{a}}_{x}\left[k\right]=\frac{a_{x}\left[k\right]-a_{x}\left[k-1\right]}{t\left[k\right]-t\left[k-1\right]}$, where ${\dot{a}}_{x}\left[0\right]=0$ and ***t*** is the vector of the timestamps. Similarly, the derivative signals, ${\dot{a}}_{y}$, ${\dot{a}}_{z}$, ${\dot{\omega }}_{x}$, ${\dot{\omega }}_{y}$, and ${\dot{\omega }}_{z}$, are computed in the same way. Given any real number α ∈ ℝ, the absolute value of α is denoted as |α|. Given any column vector ***a***_*x*_, the symbol |***a***_*x*_| denotes a new column vector obtained from the element-wise absolute value of ***a***_*x*_. When applied to a set S, the operator |·| indicates the cardinality of the set, i.e., the number of its elements |S|∈ℕ.

### 2.2. Human labeling

#### 2.2.1. Labeling strategy

The uncertainty of human gait labeling, which is widely accepted as the gold standard, is first evaluated in this paper. The datasets collected on the treadmill were labeled by *N* = 5 labelers $\left(G_{3}=\left\{S_{9},\ldots ,\;S_{13}\right\}\right)$. The uncertainty of manual labeling was quantified by studying the inter-labeller differences. The labelers were trained to use Mokka 3D Motion Kinematic & Kinetic Analyzer version 0.6.2^2^ (Barre and Armand, 2014) to identify four (*N*_*e*_ = 4) key gait events, i.e., HO, TO, HS, and FF for both left and right feet. Note that different gait terminologies are used in different works (Vu et al., 2020). In this work, the following definitions for the gait events within a stride are applied: HS is defined as the first sample at which consistent contact between the heel and the ground is achieved after the swing phase; FF is defined as the first sample at which the tip of the foot is flat on the ground after an HS event; HO is defined as the first sample at which the heel (marker) leaves the ground after an FF event; and TO is defined as the first sample at which the toe (marker) leaves the ground after a HO event.

All labelers were given the same task of labeling 12 datasets collected from four (*N*_*h*_ = 4) healthy participants *S*_1_ − *S*_4_, each participant walking at three walking speeds (1.11, 0.86, and 0.53m/s). This corresponds to roughly *N*_*L*_ = 2·*N*_*s*_·*N*_*v*_·*N*_*h*_·*N*_*e*_ = 4, 800 events manually labeled by each labeler, leading to *N*_*E*_ = *N*·*N*_*L*_ = 24, 000 labeled events obtained in total. The labels obtained from each labeler are the motion capture system timestamps of the occurrence of each gait event.

#### 2.2.2. Labeling condensation process

In general, labeling is not a difficult task for a human being, while it comes with some caveats, such as lossy marker data when the makers are disappeared for a few samples in the monitor due to marker occlusion, the lack of consistency of a labeler^3^, and the lack of consistency among different labelers^4^. Labeling uncertainty is thus to be expected, which introduces a new problem: one cannot simply compute statistics on the manual events by constructing a matrix with the labels' arrays by all labelers since

**Figure 2:**
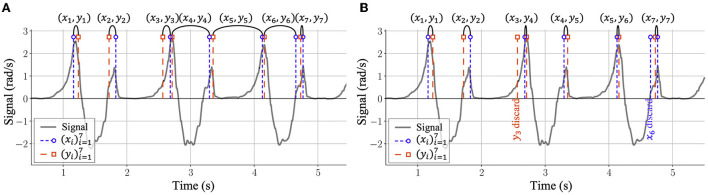
The illustration of matching algorithm for manual labeling of motion capture data. Given two label sequences ${\left(x_{i}\right)}_{i=1}^{7}$ and ${\left(y_{j}\right)}_{j=1}^{7}$ from two labelers containing the same number of labels, it is demanded to find the best sequence of index pairs (*i, j*), associating elements of *x*_*i*_ to elements of *y*_*j*_ such that one can then proceed in computing the error between sequences ${\left(x_{i}\right)}_{i=1}^{7}$ and ${\left(y_{j}\right)}_{j=1}^{7}$. **(A)** An example of matching results of two label sequences according to their index. It can be seen that the resulting fourth, fifth, and sixth pairs of labels (*x*_4_, *y*_4_), (*x*_5_, *y*_5_), and (*x*_6_, *y*_6_) do not correspond to the same gait events. **(B)** An illustration of NWA algorithm for matching labels from different human labelers. With NWA, gait event labels are matched according to their relative distances and two labels, namely *y*_3_ and *x*_6_, are considered as outliers and discarded in the further human error computation. The reasons for the existence of such outliers include human labeling errors as well as the errors from the automatic labeling algorithms.

1. there is no *a priori* guarantee that all labelers label the same number of gait events on a specific dataset;
2. even if all labelers provide the same number of labels, it does not mean that the obtained labels correspond to the same gait events. Figure 2A illustrates an example of this situation: Even if the two label sequences ${\left(x_{i}\right)}_{i=1}^{7}$ and ${\left(y_{j}\right)}_{j=1}^{7}$ from two labelers for the same dataset contain the same number of labels, the fourth (*x*_4_, *y*_4_), fifth (*x*_5_, *y*_5_), and sixth (*x*_6_, *y*_6_) pairs of labels do not correspond to the same gait events.

To evaluate the uncertainty in the manual labels for a specific gait event, each label of a labeler *S*_*i*_ needs to be first matched to the corresponding label of another labeler *S*_*j*_. This is necessary for each pair of distinct labelers (*S*_*i*_, *S*_*j*_) for (*i, j*) ∈ {9, …, 13} × {9, …, 13}, *i*≠*j*. To achieve this labels-condensation, a matching algorithm is applied iteratively among pairs of labelers until the number of labels for each labeler converges to the same number and the labels from different labelers correspond to the same sequence of gait events. This matching algorithm is conducted for each gait event, side (left or right), and experiment (walking speed and walking participant) individually.

The employed matching algorithm is the Needleman-Wunsch algorithm (NWA) (Needleman and Wunsch, 1970), which was developed for DNA sequence matching. The basic idea of NWA is to apply dynamic programming to find the optimal alignment of two label sequences while allowing for the insertion/deletion of entries from either label sequence. The NWA is implemented in Python3 with numpy. To apply the NWA, given any two label sequences ${\left(x_{i}\right)}_{i=1}^{m}$ and ${\left(y_{j}\right)}_{j=1}^{n}$ for some *m, n* ∈ ℕ from two labelers, the scoring function, including the match score (which measures the difference between two labels at the same index) and insertion/deletion score (which penalizes one insertion/deletion of a label in one of the label sequence), for the labeling condensation is defined as follows:

- Match score: *s*(*x*_*i*_, *y*_*j*_) = |*x*_*i*_ − *y*_*j*_| ;
- Insertion/deletion score: $s\left(x_{i},y_{j}\right)=\bar{E}\left({\left(\delta x_{i}\right)}_{i=1}^{m}\right)$, where δ*x*_*i*_ = *x*_*i*_ − *x*_*i*−1_ with *x*_0_ = 0.

With this scoring function, two label sequences of gait events ${\left(x_{i}\right)}_{i=1}^{m}$ and ${\left(y_{j}\right)}_{j=1}^{n}$ are optimally aligned according to NWA algorithm (Needleman and Wunsch, 1970), leading to two sequences that have the same number of labels. An illustration of the NWA matching results is shown in Figure 2B.

For every gait event, walking speed, and walking participant, the corresponding five label sequences from five labelers were matched between each other using the NWA. After this matching process, the resulting five labeler event sequences have the same number of labels can be grouped in matrices for error analysis.

#### 2.2.3. Limit of agreement

Given the matched gait event labels among five labelers, the uncertainty of the human labeling is quantified as the limit of agreement (LOA) and is assessed as follows. Let the vector ***l***_*i*_ consist of all matched labels (labels from all gait speeds and walking participants datasets) of a gait event from a labeler $S_{i}\in G_{1}$, and let $N=|G_{1}|$. A type of gait event's label uncertainty is determined by first grouping all the labels of this type of gait event across the five labelers into a matrix as:


(1) $$\begin{array}{l} L=\left(l_{1},l_{2},\ldots ,l_{N}\right)\in {\mathbb{R}}^{M\times N} \end{array}$$

where *M* is the number of matched labels for this type of gait event and *N* is the number of labelers. Next, the ground-truth labels, denoted as $\bar{l}$, of this type of gait event are computed by averaging the rows of ***L***, i.e., $\bar{l}=\frac{1}{N}\sum_{i=1}^{N}l_{i}$. Then, an error matrix ***D*** ∈ ℝ^*M*×*N*^ is computed as $D\left[:,i\right]=\bar{l}-L\left[:,i\right]$, where *i* ∈ {1, 2, …, *N*}. With the obtained matrix ***D***, the LOA is computed as the 95-th percentile of the ordered list (sorted from least to greatest) obtained from the absolute values of the entries in *D*. The ground-truth labels $\bar{l}$ are considered as a benchmark for the further validation of the proposed gait event detection algorithm.

### 2.3. Automated gait analysis

**Figure 3:**
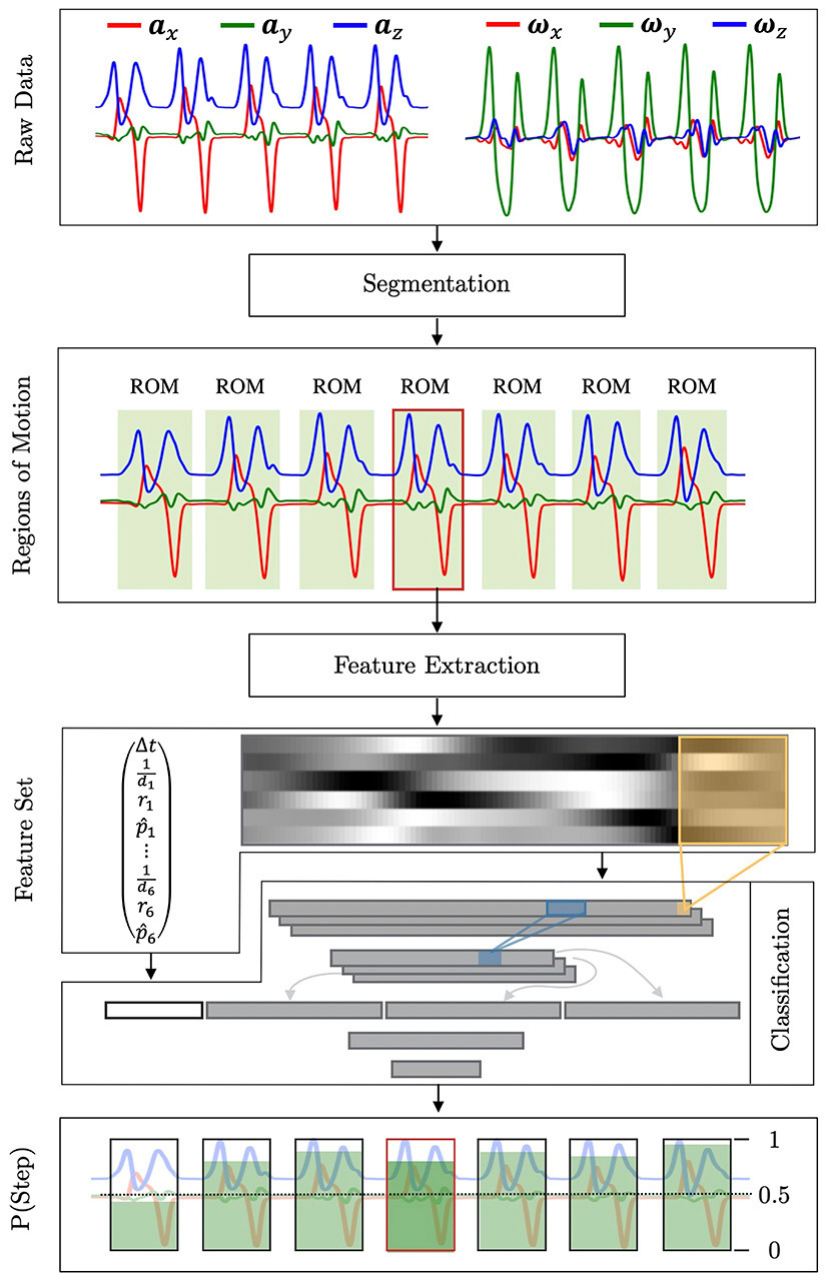
Illustration of the WGAS's automated stride detection pipeline, which takes in raw sensor data including acceleration and angular velocity, from which it detects gait strides. The figure outlines each performed operation and shows its respective result. First, the raw data is segmented into multiple ROMs, which constitutes candidate strides. Each ROM is further analyzed individually, by extracting features that are passed to the *stepperNet*, whose architecture is detailed in Section 2.3.3. Finally, the *stepperNet* outputs the probability of the input features. If the probability is larger than 0.5, the ROM is accepted as a valid stride.

This section describes the details of the proposed gait analysis algorithm. The flowchart of the algorithm is shown in Figure 3.

#### 2.3.1. Data segmentation

The potential strides are first identified within a WGAS recording by analyzing the recorded raw signals of the angular rate and acceleration. To do so, the raw signals are first segmented into multiple regions of motion (ROMs), which represent continuous foot movements and potential strides, by applying filters and dynamic thresholds. The details of the applied filters and thresholds can be found in the Supplementary material.

#### 2.3.2. Feature extraction

To classify whether an obtained ROM constitutes a stride or not, its underlying raw signals **R** = (***a***_*x*_, ***a***_*y*_, ***a***_*z*_, ***ω***_*x*_, ***ω***_*y*_, ***ω***_*z*_) are characterized by a set of features F={M,c}, consisting of a normalized and resampled IMU signal matrix, **M** ∈ ℝ^100 × 6^, and a correlation score vector, **c** ∈ ℝ^19^. **M** consists of six column vectors as $\text{M}=\left({\hat{a}}_{x},{\hat{a}}_{y},{\hat{a}}_{z},{\hat{\omega }}_{x},{\hat{\omega }}_{y},{\hat{\omega }}_{z}\right)$, where given the acceleration vector ***a***_*x*_ within the considered ROM, the column vector ${\hat{a}}_{x}$ is obtained by first min-max normalize ***a***_*x*_ to rescale its range into [0, 1], and then resample the normalized data to 100 samples by interpolation using discrete Fourier transform (Bracewell and Bracewell, 1986). The obtained **M** matrix is shown in Figure 3 as a grayscale image. The row vector **c** quantifies the similarity between the considered ROM signal **R** and the template stride signal $\text{T}=\left({\bar{a}}_{x},{\bar{a}}_{y},{\bar{a}}_{z},{\bar{\omega }}_{x},{\bar{\omega }}_{y},{\bar{\omega }}_{z}\right)$, where **T** ∈ ℝ^100 × 6^. In this work, each column vector of **T** is chosen as the average of 448 recorded stride signals, which have been resampled to 100 data points first, from four healthy participants $G_{2}=\left\{S_{5},\ldots ,\;S_{8}\right\}$. The stride template signal **T** reflects a normal stride in terms of acceleration and angular rate. To account for the temporal distortion of two signals **R** and **T**, which may be caused by different gait patterns such as walking speed, stride length, and cadence, a matching technique DTW (Berndt and Clifford, 1994; Giorgino et al., 2009) is applied to align each column of ROM signal **R** onto each column of stride template signal **T**. The rationale behind DTW is to stretch or compress the signal **R**, such that the cumulative Euclidean distance, which is referred to as DTW distance, between the resulting warped signal $\tilde{\text{R}}$ and **T** is minimized under certain constraints. After **R** has been warped to **T** along the optimal warping path, the obtained DTW distance is normalized by dividing the optimal warping path length. The normalized DTW distance for *i*-th column of **R** is denoted as *d*_*i*_, where *i* ∈ {1, 2, …, 6}. In addition, the Pearson's correlation coefficient *r*_*i*_ and its p-value *p*_*i*_ for *i*-th column of the warped signal $\tilde{\text{R}}$ and **T** are calculated to characterize the similarity of **R** and **T**. Those *p*-values of Pearson's correlation are further rescaled by ${\hat{p}}_{i}=0.1\log p_{i}$. Together with the time duration of the considered ROM signal Δ*t*, the correlation score vector **c** is constructed as $\text{c}={\left(\Delta t,\frac{1}{d_{1}},r_{1},{\hat{p}}_{1},\ldots ,\frac{1}{d_{6}},r_{6},{\hat{p}}_{6}\right)}^{⊤}$.

#### 2.3.3. Feature classification

The extracted features set F of each ROM is then fed into a neural network, which classifies if the corresponding ROM signals represents a stride or not. The architecture of the proposed CNN, *stepperNet*, is illustrated in Figure 3, which accepts F={M,c} as an input and outputs the probability of the feature set being a stride. It is composed of six total layers. The first is a convolutional layer, in which three kernels (with dimensions of 10 × 6), a step size of 1, and zero padding are applied to **M**. The resulting layer (91 × 1 × 3) is applied with a max pooling kernel (3 × 1) to compress its dimensionality. The obtained three reduced feature maps are then concatenated into a single vector (90 × 1), to which **c** is appended. The joined vector is then passed through two fully connected layers with 30 and 15 neurons each. Finally, the output of the network is normalized to a probability by the softmax function (Goodfellow et al., 2016). For any ROM signals: if the *stepperNet* outputs a probability greater than 0.5, the ROM is considered to be a walking stride, and thus the gait event detection algorithm will be further applied for intra-stride-based detection of gait events; if the stride probability is lower than 0.5, the ROM is assumed to be non-stride foot motion and will be rejected for further analysis.

#### 2.3.4. Gait event detection

**Figure 4:**
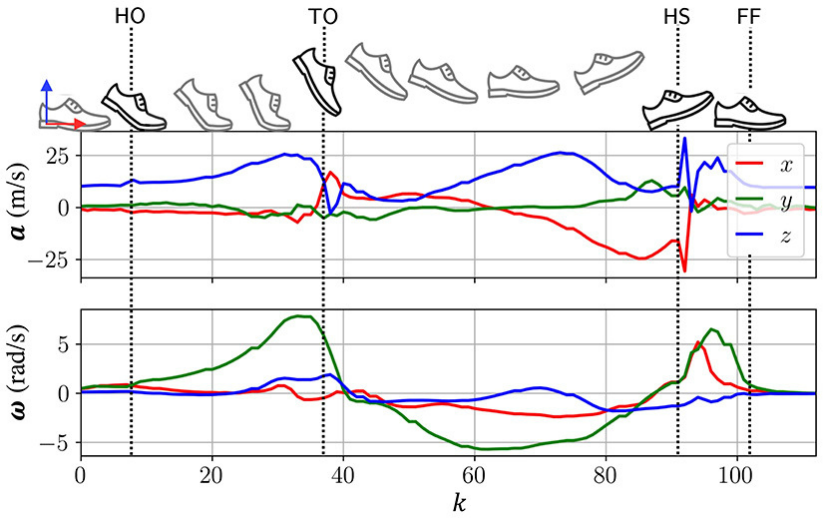
An example of the measured acceleration and angular rate over the course of a stride. Four gait events, HO, TO, HS, and FF, are identified by the manual labels obtained from the motion capture system.

For any ROM, which has been determined to constitute a stride, the four gait events HO, TO, HS, and FF are identified by event-specific features in the recorded acceleration and angular rate within the signal region of the ROM. The correspondence of these features to the occurrence of the event was established experimentally by comparing the WGAS recordings to the manual labeled events of the accompanying motion capture system measurements obtained from Section 2.2. Figure 4 shows an example of the raw acceleration and angular rate over a stride recorded by the WGAS's IMU, together with the location of the gait events.

It can be observed that between the HO and FF, the foot can be assumed to be moving. As both gait events are the result of changes in the foot's orientation, in this work, HO and FF are detected by taking the first and last time stamp where ***ω***_*a*_[*k*]/σ_***ω***_*a*__>0.5, where $\omega_{a}\left[k\right]=\sqrt{{\left(\omega_{x}\left[k\right]\right)}^{2}+{\left(\omega_{y}\left[k\right]\right)}^{2}+{\left(\omega_{z}\left[k\right]\right)}^{2}}$ and σ_***ω***_*a*__ is the standard deviation of the column vector ***ω***_*a*_. As the HS event resembles an impulse that manifests itself in an abrupt change in the measured acceleration and its impact is markedly experienced in the *z*−direction, the HS event is determined by the location of the most prominent peak in ${\dot{a}}_{z}$. By comparing the WGAS's measurements to the manual labels of the motion capture system the TO event is determined as the first timestamp, where ***a***_*x*_[*k*] = ***a***_*z*_[*k*].

#### 2.3.5. Data synchronization

The validation dataset consists of two WGAS measurements (left and right) with a corresponding motion capture system recording, each of which is logged with its own internal clocks. To compare the manually labeled gait events to the events detected by the gait event detection algorithm, we first ensure that they are temporally synchronized. To this end, each WGAS is set to initiate a vibration sequence once they are turned on, vibrating for 1*s*, a 1*s* pause, and a second vibration of 1*s*. As the experiment starts with the participant standing on the treadmill of the motion capture system the vibration sequence is also picked up by the treadmill's integrated force plate. This allows for the temporal synchronization between the data collected by the two systems.

The proposed algorithms of gait stride detection and gait event detection are implemented in Python3. Further important tools include PyTorch, which was used as the machine-learning framework (Paszke et al., 2019) and the dtw-python library as the DTW library (Giorgino et al., 2009). Lastly, the proposed algorithms extensively use various toolboxes of the SciPy library (Virtanen et al., 2020).

## 3. Results

### 3.1. Uncertainty of human labeling

For all labeled datasets, the label matching results of the NWA algorithm are presented in Table 1. It can be seen that only a small percentage (1.49%) of labels were discarded for all gait events. Overall, 413 labels were discarded from a total of 27,683 manually labeled events. The maximum number of discarded events was 23 out of 333 available (for HO event of participant *S*_12_ with walking speed 1.11 m/s).

**Table 1:** Labels matching results obtained by applying the NWA across the five labelers's manual labeling of motion capture system data.

**Event**	**Labels**	**Removed**	**Percentage**
HO	6,916	121	1.75
TO	6,930	95	1.37
HS	6,924	84	1.21
FF	6,913	113	1.63
Total	27,683	413	1.49

**Figure 5:**
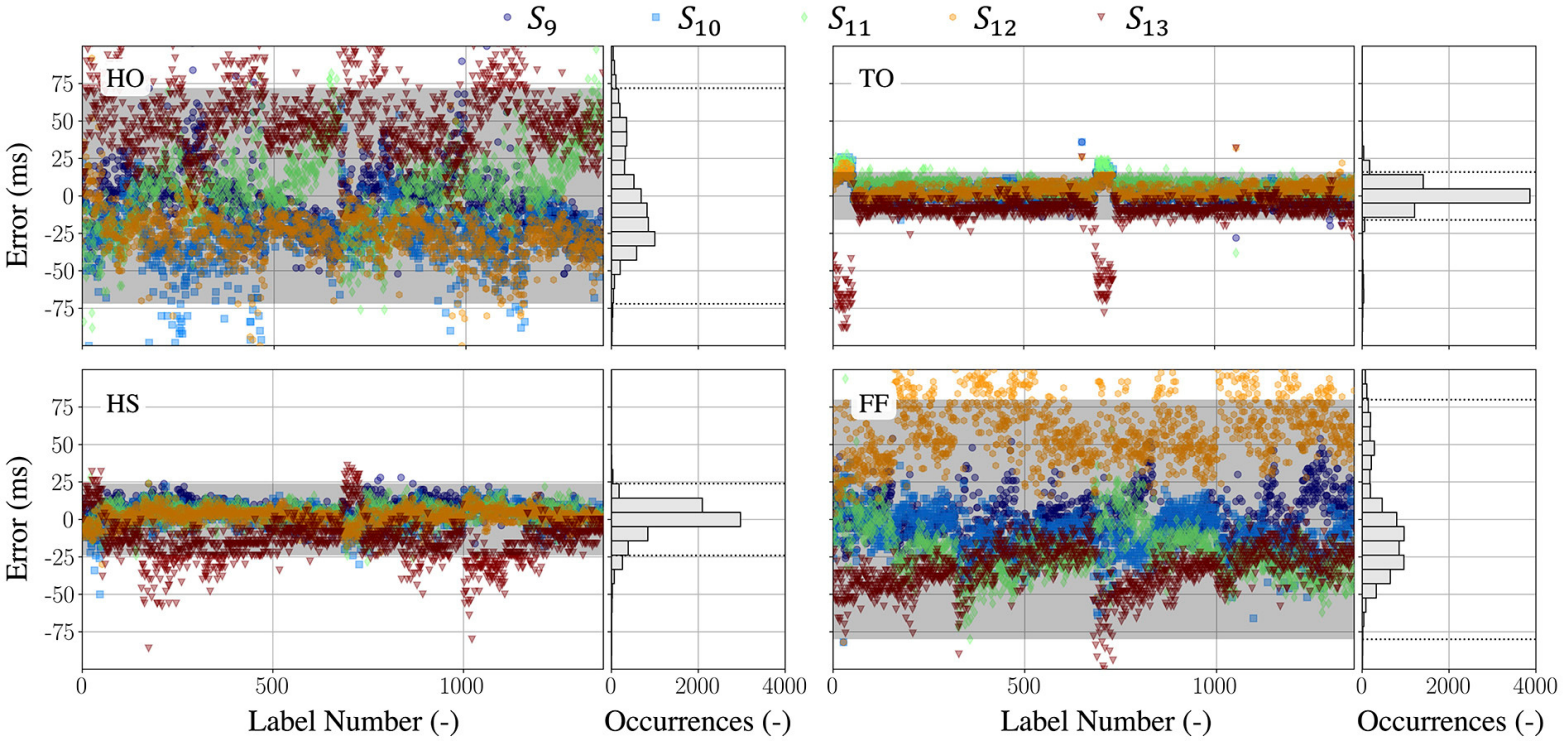
The error distributions and the LOA of the labels for four gait events HO, TO, HS, and FF. The LOA in this work for HO, TO, HS, and FF are 72*ms*, 16*ms*, 24*ms*, and 80*ms* respectively. In each subplot: the left plot shows all identified manual labels from five labelers *S*_9_ − *S*_13_ as deviation from the average label—the gray area is the LOA band; the right plot shows the distribution of all label errors—it can be understood as a projection of all samples of the left plot onto the *y*−axis. The dotted lines in the histogram mark the LOA band.

After the procedure of label condensation, the LOA results illustrated in Figure 5 are obtained. The LOA in this work for HO, TO, HS, and FF are 72, 16, 24, and 80*ms*, respectively, which corresponds to 2–8 samples of uncertainty/disagreement when sampling at 100 Hz. It can be seen that for the main gait events HS and TO, the labels agree well among labelers as the LOA are 16 and 20*ms*, which means that only two samples on average differ between the five labelers. However, the human labeling uncertainty for the secondary gait events HO and FF reached 72 and 80*ms*, i.e., nearly eight samples of variations are observed between the labels labeled by the five labelers, which are higher than the LOA of the HS and TO labels. This is due to the intrinsic difficulty that HO and FF come within their definitions, and consequent difficulties for humans to decide from the motion capture system data whether an event of these types has occurred. As a consequence, the limits in terms of accuracy claims of automated systems are to be set around the identified LOA, i.e., the LOA is the limit of accuracy for the validated system as it represents the intrinsic uncertainty in the ground-truth measurements.

### 3.2. Gait cycle segmentation

The second dataset collected for building *stepperNet* consisted of 4,014 labeled feature sets, of which 1,888 represented strides, and the rest characterized non-stride foot movement. For training *stepperNet*, the collected data were randomly split into a training dataset (3,493 features), with which the network's parameters were optimized, and a test set (521 features) which was used to monitor *stepperNet*'s performance in training.

**Figure 6:**
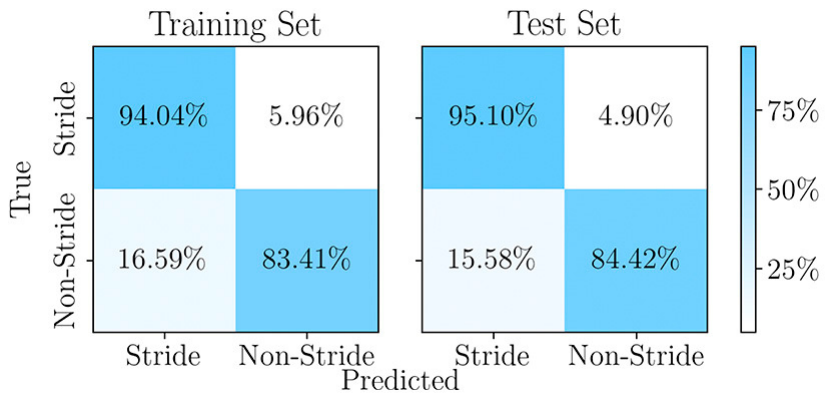
The performance of *stepperNet* at the conclusion of its training on the training dataset and the separate test dataset. The residual loss on the two datasets is 4.26 × 10^−3^ and 4.02 × 10^−3^ respectively.

The *stepperNet* was trained by stochastic gradient descent (Bottou, 2010) over 7 epochs, with the learning rate and momentum set to 0.015 and 0.75, respectively. Due to the small network size and the number of training examples, the performance of the trained network is highly dependent on the initially chosen weights and biases. To optimize the stride classification capabilities of *stepperNet*, the training procedure was repeated multiple times, each time with different random initial internal parameters, and the highest scoring network was saved and deployed. The optimized *stepperNet*'s performance on the training and validation data are reported in Figure 6, with stride and non-stride detection accuracy of 95.10% and 84.42%, respectively.

### 3.3. Gait event detection

To evaluate the performance of the gait event detection algorithm, the timestamp of each type of gait event detected by the proposed algorithm is compared to the ground truth labels, i.e., the timestamp of the gait event identified manually in the motion capture system data. Note that the ground truth labels obtained from Section 2.2 are served as the baseline for the validation of this gait event detection algorithm. The performance of the gait event detection algorithm is quantified by four error metrics for each type of gait event, i.e., the mean error, the mean absolute error (MAE), the root mean squared error (RMSE), and the 95-th percentile error (LOA). The overall error metrics for the detected HO, TO, HS and FF, averaged across all participants and treadmill speeds, are presented in Table 2. In total, the timestamps of the WGAS detected gait events for about 1,350 strides are compared to the ground truth labels. This number has a slight differences for different gait events as shown in Table 2, this is due to the reason that the manual labels of a gait event that are not identified by all labelers has been discarded in the labeling condensation process.

**Table 2:** Error metrics for WGAS gait event detection results.

**Event**	**Mean (ms)**	**STD (ms)**	**MAE (ms)**	**RMSE (ms)**	**LOA (ms)**	**Samples (-)**
HO	9	29	24	31	60	1,338
TO	2	7	5	7	10	1,347
HS	-2	16	9	16	20	1,355
FF	9	34	13	36	30	1,349

**Figure 7:**
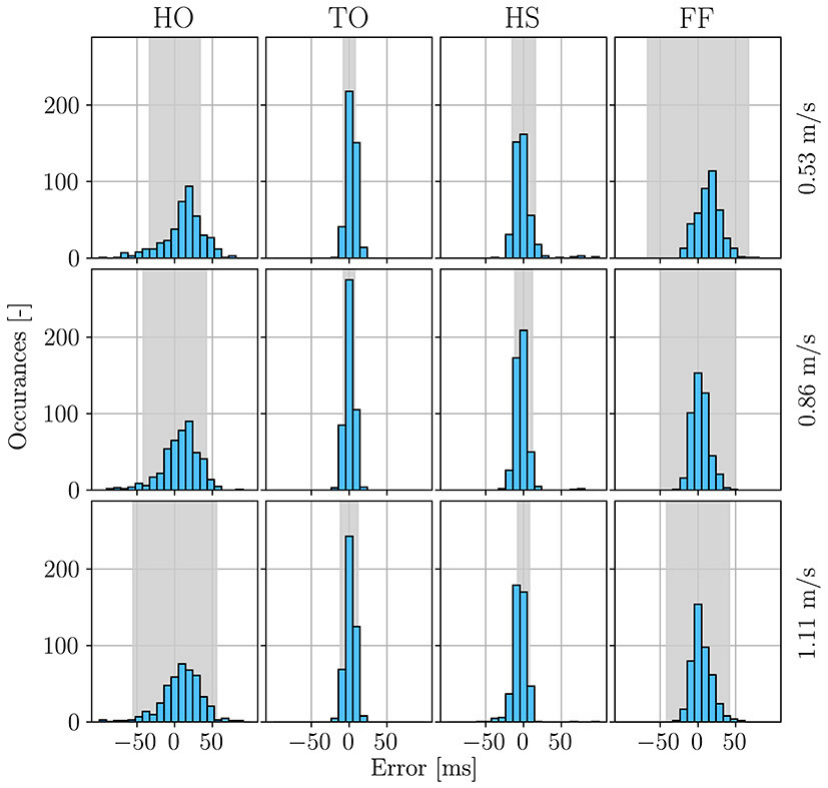
Error histograms for the WGAS gait event detection errors grouped by the set treadmill speed and event type. The histograms are plotted with a bin size of 10*ms*, and for comparison, the uncertainty bounds of the manual labeled events are shown in gray.

The error metrics are also presented for each event averaged across all participants but grouped by treadmill speed as shown in Figure 7 and Supplementary Table S1. It can be observed that as the walking speed increases from 0.53 to 1.11 m/s, the event detection error of the algorithm appears to slightly decrease (HO: from 11 ± 22 ms to 8 ± 27 ms, TO: from 3 ± 7 ms to 1 ± 6 ms, HS: from −5 ± 14 ms to −4 ± 13 ms, FF: from 15 ± 17 ms to 6 ± 12 ms). This can be explained by the reason that with the higher walking speed, the event features are more pronounced and unambiguous within the signals. As this effect is negligible, the gait event detection algorithm can be considered invariant and robust toward changes in gait speed.

Generally, it should be stated that it is difficult to discern if an error is the result of faulty event detection of the algorithm or due to the subjective nature of manual labeling. For the HO and FF, this is especially true, as the events are not definitively identifiable in the motion capture system data by eye introducing an error in the ground truth itself. An infinite number of labelers may average out every individual labeling error, but due to time and cost constraints, the limited number of labelers in this study and in practice, the ground truth may still be imperfect.

## 4. Discussion

Manual labeling of four gait events HO, TO, HS, and FF on motion capture system datasets are evaluated in this work as they are essential for analyzing temporal gait parameters, such as stride time, swing time, stance time, and single- and double-support time. As can be seen from Figure 5, The manual labels that were labeled by five labelers with the similar labeling experience exhibit a remarkable consistency among different labelers for two main gait events, HS and TO, with only two samples (around 20 ms) of variations are observed. However, they showed greater disagreement on the secondary gait events HO and FF, with up to eight samples of average variations are observed on these two gait events. This disagreement is due to the inherent challenges of observing HO and FF in optical data from motion capture systems and the fact that different labelers have different perspectives even though they have similar labeling experience. It can also been observed from the Figure 5 that even for the same labeler, such as S13, they have a large labeling deviation along the sequence of data labeling, varying from 2 to 8 samples. It is the intrinsic human labeling uncertainty regardless of human experience of labeling. This uncertainty represents the accuracy limit of human gait-labeling based on motion capture data, and it determines the limit for any validation study which considers the motion capture system as the gold standard. Low consistency between labelers can have a direct impact on gait event detection reliability. This further affects the estimation accuracy of spatio-temporal gait parameters, as well as the medical decision-making and therapy by clinicians.

The reasons for identifying HS, TO, HO, and FF gait events in a gait cycle are discussed as follows. The gait cycles can be delimited by the HS event, which is considered of paramount functional importance marking the shift from flexors to anti-gravity muscles and is also reliably detectable (Burnfield, 2010). To calculate gait parameters for each gait cycle, the movement data are interpolated from HS to the next ipsilateral HS. Errors in HS detection lead to normalization artifacts that can affect the whole gait cycle and hence all calculated parameters. TO delineates the two main phases of gait: stance and swing. Kinematic parameters are frequently reported for these phases separately, hence errors in TO detection affect not only values at TO but also parameters calculated for the phases, e.g., “knee range of motion during swing.” At both time points, parameters are frequently reported that are in phases of rapid change, such as sagittal ankle and knee angle at TO (both in the range of 100–300°/s in dependence on walking speed), or foot elevation angle at HS (Mentiplay et al., 2018). Concerning the gait events HO and FF, there is a growing interest in the importance of these phases that depict a first reaction to a state change from swing-to-stance (Nolan and Yarossi, 2011) and the preparatory phase from stance-to-swing (Lewek et al., 2018; Roelker et al., 2019). Detecting these additional gait events also allows the calculation of parameters related to push off and initial contact. These events do not correspond with traditional clinical phases of gait, however present a technical method to acquire information about critical states. Overall, more detailed segmentation of the gait cycle and calculation of movement parameters that provide a relationship to typical recovery patterns in clinical populations allows clinicians to make more objective and detailed assessments of the deviation in pathological gait and the improvements in rehabilitation progress. Furthermore, gait event detection is also critical for applications in which gait events are served to trigger assistive devices, as well as when considering the use of orthotic or therapeutic interventions, particularly in functional electrical stimulation (Zahradka et al., 2020).

After the manual labels obtained on the motion capture data, a *stepperNet* to automatically identify strides and reject non-strides from long-term walking signals is developed, and a gait event detection algorithm was adopted to segment the identified strides into sub-phases for further estimation of gait parameters. As shown in Figure 6, the stride classifier *stepperNet* shows a good classification performance with an accuracy of 94.04% in stride detection. For non-stride detection, a relatively low accuracy of 83.41% was obtained. This could be due to the fact that in the motion activity of healthy people, stride motion include only walking movements, while non-stride motion include various human motions, such as jumping, sitting and standing, climbing, etc. The variability of stride signals are much higher than the variability of non-stride signals. It can also been noticed that the classification accuracy of the test set is slightly higher than that of the training set (around 1%), this can be explained by the randomness when splitting the total feature set, which yielded marginally 1% more difficult examples in the training set than in the test set. Note that the CNN is only used for identifying a valid stride and eliminating non-stride data from the dataset that was acquired over a long (walking) period. It shall be noted that whilst one is walking for longer periods in real-life scenarios not all motions are actually strides, e.g., walking usually features breaks and motions associated to breaks such as pivoting on the spot, which are not to be considered strides/walking. The presented hybrid model allows to split the long-term “walking” data into “stride” and “non-stride” data slices, which makes further data processing (gait event) easier.

After stride segmentation, gait events are afterwards detected within the region of this identified stride by searching for specific signal characteristics. The detected gait events are validated with the ground-truth labels obtained from the manual labeling of motion capture system data. It can be seen from Table 2, the gait event detection algorithm perform well for all four gait events (HO, TO, HS, and FF), with a mean difference of less than one samples and a standard deviation of less than four samples compared to the benchmark (i.e., the labels from the motion capture system). The errors of gait events HO and FF are slightly higher than those of TO and HS. This is due to the reason that at the gait event of HO and FF (Figure 4), the signals are flat, the amplitude or the rate fluctuations of the motion signal are not as pronounced as in the TO and HS. This agrees with the results of human labeling in Figure 5, where the uncertainties of HO and FF are higher than that of TO and HS. Besides, comparing the LOA of the detected gait events, as shown in Table 2, with those of human labeling in Figure 5, it can be seen that the gait event detection algorithm are generally consistent in their predictions. Table 3 compares the results of this paper to the work reported in the literature in terms of MAE. This comparison focuses on studies with healthy participants, no pathological patients were involved in the experiments. The comparison shows that the proposed algorithm has a comparable performance in detecting HS and TO. TO has the lowest MAE compared to the other studies, while the MAE of HS is slightly higher MAE than the study of Fadillioglu et al. (2020) and Mo and Chow (2018). However, the MAE of HS is less than one sampling time (10 ms), this difference is still within the acceptable range.

**Table 3:** A comparison of the MAE for HS and TO gait event detection in healthy participants with those reported in the literature.

**Study**	**Sensor location**	**Walking condition**	**HS (ms)**	**TO (ms)**
Tiwari and Joshi (2020)	Foot	Over-ground walking	22.87	10.42
Fadillioglu et al. (2020)	Shank	Treadmill walking	7	9
Flood et al. (2019)	Waist and shank	Treadmill walking	16.41	39.84
Nazmi et al. (2019)	Foot	Treadmill walking	35	49
Mo and Chow (2018)	Pelvis	10-m walkway walking	6.2	20.3
Godiyal et al. (2018)	Thigh	Over-ground walking	9.66	16.99
**Ours**	**Foot**	**Treadmill walking**	**9**	**5**

Opposed to the commonly used approach in the literature, which straightly scans the entire walking recording to identify the gait events and then employs the gait event to define a stride, the CNN eliminates all non-stride data and only keeps stride data for further gait event detection. The main advantage of this approach is robustness, as the aforementioned method scans the entire dataset, erroneously detected gait events, have the potential to invalidate the entire analysis. Errors in our proposed method however only affect an individual stride and the detection of gait events belonging to other strides is undisturbed.

This work is based on a limited number and type of validation participants, but it still provides important insight on analyzing gait in a robust and efficient way. Furthermore, the selection of manual labeling of the motion capture data as the ground truth induces caveats. Vertical ground reaction force, specifically, was not used in this study due to a large amount of foot strikes that loaded both force plates. These events would have needed to be excluded, which would have introduced a bias into the data set. Therefore, group-based manual labeling was performed, which is also representative of how pathological gait is frequently segmented. Though males and females differ from a biomechanical perspective due to their differences in joints-relative position, the proposed system is believed to be agnostic with regard to these differences. This is because that different joint positions and body dimensions affect the motion metrics at the extremities, which is where the proposed system performs the measurements. Therefore, the motion captured by the WGAS system is not subject to potential gender-related effects. While the issue of gender balance and pathological gait have to be addressed in the future for assessing the generalizability and effectiveness of the proposed method for pathological subjects. In this study, the stride template was generated by averaging a set of healthy strides. For pathological gait, strides will not be as homogeneous, new methodologies have to be developed for optimizing the stride template, such as generating template from kernel density estimation or Gaussian mixture model, constructing a library of stride templates from different pathologies or experimental conditions. In this work, *stepperNet* is only validated on treadmill walking data, one participant's stride is likely to be identical during the same speed session. As the *stepperNet* is primarily designed for identifying walking strides and eliminating the non-walking strides data from long-term free walking data to reduce the computational cost, the performance of *stepperNet* on long-term free walking in the real-world must be validated. Future work will focus on investigating the detection accuracy of *stepperNet* in various conditions, including variable speed walking, free-range walking, and various clinical populations.

## 5. Conclusion

We described a method to estimate and analyze the uncertainty in manual labeling of human walking data from motion capture systems, which are commonly used as a benchmark. Human manual labeling uncertainty was assessed by measuring inter-labeler inconsistencies. The limits of detection for key gait events, HO, TO, HS, and FF have been estimated as 72, 16, 24, and 80*ms* respectively with a sampling time of 10 ms. Those inherent label uncertainty of human gait labeling based on motion capture data is the accuracy limit for any validation research using this technology.

Using the manual labels (obtained by averaging across all labelers) as a baseline, we also present a novel CNN-based algorithm capable of detecting key gait events with a detection error less than 25 ms in healthy, fixed speed treadmill walking. The algorithm provides an improvement on current solutions in that it allows the accurate processing of long continuous walking data streams. Clinically, the accurate detection of these events reduce distortion effects when calculating gait parameters, providing a gateway to reliable parameter calculation that are lower than the minimal clinically important difference. Achieving a high accuracy in gait parameters is paramount for empowering clinical decision-making based on mobile gait analysis.

## Data availability statement

The original contributions presented in the study are included in the article/Supplementary material, further inquiries can be directed to the corresponding author.

## Ethics statement

The studies involving human participants were reviewed and approved by the Swiss Ethics Committee (BASEC Nr Req-2019-00715) following the Good Clinical Practice Guidelines and the Declaration of Helsinki from. The patients/participants provided their written informed consent to participate in this study.

## Author contributions

JW and AS conceived and designed the study. JW, HM, AS, and BB performed the experiment and analysis. CA and GC collected the data. JW took the lead in writing the manuscript with input from HM. AS, BB, and JW was in charge of overall planning. OE, SP, and BN acquired the funding of the study. All authors provided critical feedback and helped shape the manuscript.

## Footnotes

1 https://www.freertos.org

2 https://github.com/Biomechanical-ToolKit/Mokka

3 Even if the same labeler labels the same dataset several times in the same way, a different labeling sequence will be obtained each time.

4 Even if different labelers label the same dataset with the same labeling rules, different labeling sequences will be obtained for each labeler.
