# Supplementary material for: Human gait-labeling uncertainty and a hybrid model for gait segmentation
Source: Front Neurosci. 2022 Dec 8;16:976594. doi: 10.3389/fnins.2022.976594 (PMC9773262; doi:10.3389/fnins.2022.976594)
Supplement: Supplementary file 1 [file Data_Sheet_1.PDF]

frontiers

Supplementary Material

1 DATA SEGMENTATION

To segment a data recording of WGAS into multiple ROMs that contains foot movements, a few steps of signal preprocessing are conducted as follows. A set of four mean absolute signals, $\boldsymbol{a}_m, \dot{\boldsymbol{a}}_m, \boldsymbol{\omega}_m, \dot{\boldsymbol{\omega}}_m$ are first constructed by $\boldsymbol{a}_m = \frac{1}{3}(|\boldsymbol{a}_x| + |\boldsymbol{a}_y| + |\boldsymbol{a}_z|)$, $\dot{\boldsymbol{a}}_m = \frac{1}{3}(|\dot{\boldsymbol{a}}_x| + |\dot{\boldsymbol{a}}_y| + |\dot{\boldsymbol{a}}_z|)$, $\boldsymbol{\omega}_m = \frac{1}{3}(|\boldsymbol{\omega}_x| + |\boldsymbol{\omega}_y| + |\boldsymbol{\omega}_z|)$, and $\dot{\boldsymbol{\omega}}_m = \frac{1}{3}(|\dot{\boldsymbol{\omega}}_x| + |\dot{\boldsymbol{\omega}}_y| + |\dot{\boldsymbol{\omega}}_z|)$, respectively. In the first segmentation step, the recording is segmented by scanning for coherent sequences that satisfy the following conditions:

1. $f_{lp}(\dot{\boldsymbol{a}}_m, 0.75\,\mathrm{Hz}) > 1\,\mathrm{m/s^3}$;
2. $\Delta t > 0.25\,\mathrm{s}$;

where $f_{lp}(\dot{\boldsymbol{a}}_m, f_c)$ denotes a low-pass filter with a cut-off frequency f_c applied to the signal $\dot{\boldsymbol{a}}_m$, Δt denotes the duration of the signal that satisfies the first condition. These two conditions ensure that the resulting data segment contains enough motion and the duration of the region is long enough to warrant further analysis.

In the second segmentation step, the obtained signals $\boldsymbol{a}_m$ and $\boldsymbol{\omega}_m$ from the first segmentation step is split further by applying region-specific thresholds $\theta_{\boldsymbol{a}_m}$ and $\theta_{\boldsymbol{\omega}_m}$ defined as follows:

$$\theta_{\boldsymbol{a}_m} = \beta_{\boldsymbol{a}_m} + \alpha_{\boldsymbol{a}_m} \left(\bar{\mathbb{E}} \left(\boldsymbol{a}_m \right) - \beta_{\boldsymbol{a}_m} \right), \tag{S1}$$

$$\theta_{\boldsymbol{\omega}_m} = \beta_{\boldsymbol{\omega}_m} + \alpha_{\boldsymbol{\omega}_m} \left(\bar{\mathbb{E}} \left(\boldsymbol{\omega}_m \right) - \beta_{\boldsymbol{\omega}_m} \right), \tag{S2}$$

where $\alpha_{\boldsymbol{a}_m}$ and $\alpha_{\boldsymbol{\omega}_m}$ are ROM tuning parameters, $\beta_{\boldsymbol{a}_m}$ and $\beta_{\boldsymbol{\omega}_m}$ refer to the baseline of $\boldsymbol{a}_m$ and $\boldsymbol{\omega}_m$, which are estimated using iterative polynomial fitting on the given $\boldsymbol{a}_m$ and $\boldsymbol{\omega}_m$ (Gan et al., 2006). By thresholding $\boldsymbol{a}_m$ and $\boldsymbol{\omega}_m$ against their respective thresholds, $\theta_{\boldsymbol{a}_m}$ and $\theta_{\boldsymbol{\omega}_m}$, two binary column vectors $\boldsymbol{a}_b$ and $\boldsymbol{\omega}_b$ are constructed by

$$\boldsymbol{a}_b[k] = \begin{cases} 1, & \text{if } \boldsymbol{a}_m[k] > \theta_{\boldsymbol{a}_m} \\ 0, & \text{else} \end{cases} \tag{S3}$$

$$\boldsymbol{\omega}_b[k] = \begin{cases} 1, & \text{if } \boldsymbol{\omega}_m[k] > \theta_{\boldsymbol{\omega}_m} \\ 0, & \text{else} \end{cases} . \tag{S4}$$

With those binary vectors $\boldsymbol{a}_b$ and $\boldsymbol{\omega}_b$, each data segment obtained from the first segmentation step is further divided into continuous regions that satisfy the condition $f_{lp}(\boldsymbol{a}_b + \boldsymbol{\omega}_b, 4\,\mathrm{Hz}) > 1.15$. Lastly, to ensure the segmentation procedure only yields relevant data segments, which represent candidate strides, any ROM are discarded that do no meet $\bar{\mathbb{E}}(\dot{\boldsymbol{a}}_m) > 10\,\mathrm{m/s^3}$ and $\bar{\mathbb{E}}(\dot{\boldsymbol{\omega}}_m) > 0.8\,\mathrm{rad/s^2}$. The values of all thresholds used in this work are determined based on processed datasets, they do change for new subjects.

2 GAIT EVENT DETECTION

REFERENCES

Gan, F., Ruan, G., and Mo, J. (2006). Baseline correction by improved iterative polynomial fitting with automatic threshold. *Chemometrics and Intelligent Laboratory Systems* 82, 59–65

		T012			T013			T014			T015			Total
		0.53 m/s	0.86 m/s	1.11 m/s	0.53 m/s	0.86 m/s	1.11 m/s	0.53 m/s	0.86 m/s	1.11 m/s	0.53 m/s	0.86 m/s	1.11 m/s	
HO	Mean	-17	-10	4	34	28	7	9	-8	-3	17	18	24	9
	STD	38	25	30	20	24	35	19	31	27	10	14	15	29
	MAE	32	20	23	31	31	28	18	21	18	18	19	24	24
	RSME	41	27	30	39	37	36	21	32	27	20	23	28	31
	LOA	70	50	50	60	60	80	40	70	70	30	40	50	60
TO	Mean	4	-1	-4	4	1	2	5	4	4	-1	-2	3	2
	STD	6	6	6	8	7	6	6	6	6	7	6	8	7
	MAE	5	4	5	6	5	4	6	5	5	4	4	6	5
	RSME	7	6	8	9	7	6	8	7	7	7	7	8	7
	LOA	10	10	10	20	10	10	10	10	10	10	10	20	10
HS	Mean	5	-5	-9	-1	1	-6	-8	-5	-6	1	-1	0	-3
	STD	15	8	8	10	25	15	6	7	19	27	13	10	16
	MAE	8	7	9	8	11	9	8	5	10	14	7	6	9
	RSME	16	9	12	10	25	16	10	8	20	27	13	10	16
	LOA	20	20	20	20	30	40	20	20	20	80	20	20	20
FF	Mean	15	4	5	11	7	-2	14	8	4	21	5	13	9
	STD	19	13	13	14	115	9	21	13	12	12	11	16	34
	MAE	19	10	10	14	19	7	19	11	9	22	9	16	13
	RSME	24	14	14	18	116	10	25	15	12	25	12	21	36
	LOA	40	30	30	30	20	20	50	30	20	40	30	40	30

Table S1. Automatic gait analysis detailed error statistics. All values are in ms.
